# The mismatch between morphological and molecular attribution of three *Glossogobius* species in the Mekong Delta

**DOI:** 10.1186/s40850-022-00137-6

**Published:** 2022-06-23

**Authors:** Ngon T. Truong, Gieo H. Phan, Tran T. H. Lam, Ton H. D. Nguyen, Do T. Khang, Men T. Tran, Nam S. Tran, Quang M. Dinh

**Affiliations:** 1 Department of Molecular Biotechnology, Biotechnology Research and Development Institute, Can Tho University, Xuan Khanh Ward, Ninh Kieu District, Can Tho, 900000 Vietnam; 2 Faculty of Agriculture and Rural Development, Kien Giang University, Minh Luong Town, Chau Thanh District, Kien Giang, 920000 Vietnam; 3 Institute of High Quality Biotechnology - Food Technology, Cuu Long University, National Road 1A, Phu Quoi Ward, Long Ho District, Vinh Long, 850000 Vietnam; 4 Department of Biology, School of Education, Can Tho University, Xuan Khanh Ward, Ninh Kieu District, Can Tho, 900000 Vietnam; 5 Department of Biology, College of Natural Science, Can Tho University, Xuan Khanh Ward, Ninh Kieu District, Can Tho, 900000 Vietnam; 6 Department of Environmental Sciences, College of Environment and Natural Resources, Can Tho University, Can Tho, 900000 Vietnam

**Keywords:** Goby, *Glossogobius* genus, Genetic distance, Phylogenetic tree, Vietnam

## Abstract

**Background:**

The Vietnamese Mekong Delta (VMD) is the granary for the whole country, providing animal and plant resources, especially fish. Among the fish species, the genus *Glossogobius* are the majority. Until now, research for this species has been solely relied on fish morphology for identification. Hence, the present study aimed to describe the morphological variations of the morphologically identified gobies and to validate them at the molecular level through the sequencing of the barcode region, the mitochondrial cytochrome C oxidase subunit I (COI) gene to preliminary provide fundamental information for conservation.

**Results:**

The mitochondrial cytochrome C oxidase subunit I genes were amplified successfully with an approximate size of 650-680 bp. Their morphometries were quite different, and the genetic distance (*p*-value) among groups and within groups ranged from 0.00 to 0.12. The similarity of the COI gene sequences between the analyzed samples and in the NCBI database was from 87.01 to 100%. The specimens of *G. aureus*, *G. giuris* and *G. sparsipapillus* were interspersed in small branches of the phylogenetic tree with a low genetic distance highlighting that the genetic diversity of COI gene was low among species. Therefore, it is recommended that a combination of morphological method and mtCOI DNA barcoding is required for accurate classification.

**Conclusion:**

This study helps determine three distinct lineages of *Glossogobius* species, so an appropriate strategy can be proposed for exploitation and conservation.

**Supplementary Information:**

The online version contains supplementary material available at 10.1186/s40850-022-00137-6.

## Background

The Mekong Delta region encompasses a large portion of south-western Vietnam of over 40,500 km^2^ and is covered by water depending on the season. The wet coastal geography makes the region an essential source of agriculture and aquaculture products for the whole country [1]. Not only famous for being a large granary, the Mekong Delta is also considered a source of genetic diversity, presenting various living organisms, especially gobies, which are among the most common species [2, 3].

The *Glossogobius* spp. are the primary source of protein for local residents in the Mekong Delta and constitutes the central part of the diet in different cultures, and they also play an essential role in the local economy, specifically providing jobs and investment opportunities for many countries [4]. The morphometrics and meristics of *Glossogobius* spp. in the Mekong Delta, including *G. giuris*, *G. aureus* and *G. sparsipapillus*, has been found to change with ecoregions along the riverine [5, 6] and coastline regions [7–14]. However, this morphological variation could be due to the environmental adaptation or genetics. Genetic variation is the raw material in a species and population, enabling them to adapt to changes in their environment. This study, therefore, aimed to describe the morphological variations of the identified gobies and to validate them at the molecular level through the sequencing of the barcode region, the mitochondrial cytochrome C oxidase subunit I (COI) gene to preliminary provide fundamental information for conservation.

## Methods

### Study site, fish collection and analysis

**Fig. 1:**
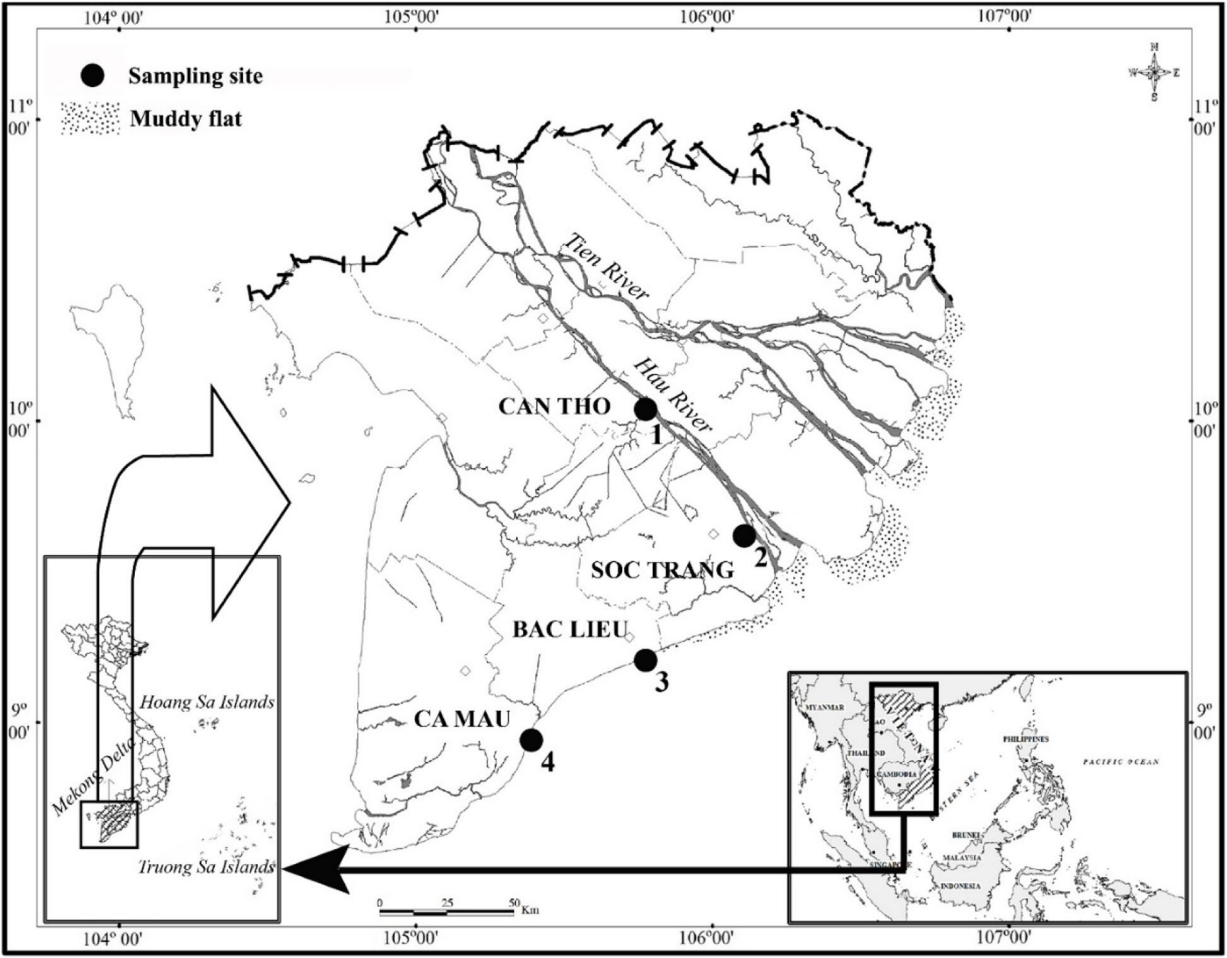
The sampling map modified from Fig. 1 of Dinh [15] (•: Collection sites; 1: Cai Rang, Can Tho; 2: Long Phu, Soc Trang; 3: Hoa Binh, Bac Lieu; 4: Dam Doi, Ca Mau)

This research was carried out at four sites along the riverine to estuarine and coastline ecoregions, including Cai Rang in Can Tho (CRCT), Long Phu in Soc Trang (LPST), Hoa Binh in Bac Lieu (HBBL), and Dam Doi in Ca Mau (DDCM) (Fig. 1). These regions are characterized by a semi-diurnal tidal range of ~ 1.2 m, a temperature of ~ 27 °C, pH of ~ 8, and salinity of ~ 12 ‰ in LPST and 0‰ in CRCT. It rarely rains in the dry season (from January to May) but rains heavily almost every month in the wet season (from June to December), with an average monthly rainfall of 400 mm [1, 16].

Fish specimens were collected monthly from 01/2020 to 12/2020 using trawl nets with a 1.5 cm mesh aperture at the cod-end. After 2–3 h setting at the highest tide in each study site, nets were retrieved to collect fish specimens which were then identified based on their external description [17]. Thereafter, twelve separate samples representing three morphological species at four sampling sites were stored in 96% ethanol for DNA extraction. A total of 742 *G. aureus*, 1291 *G. giuris* and 764 *G. sparsipapillus* used for morphological analysis were observed and the external morphological traits of the three fish species were recorded before fish were euthanized with MS222 and fixed with 5% formalin solution for other experiments (Animal Welfare Assessment BQ2020–02/KSP). The total length (TL), body depth (BD), head length (HL), eye diameter (ED), the distance between eyes (DE), specimen weight and morphometrics traits such as ED/HL, DE/HL, BD/TL, HL/TL were measured at the laboratory.

### DNA extraction and polymerase chain reaction (PCR)

The genomic DNA from the twelve specimens was extracted following the method of Rogers and Bendich [18]. The DNA amplification was carried out at the Laboratory of Molecular Biology of the Biotechnology Research and Development Institute, Can Tho University, based on the research methods of Nguyen and Duong [19]. One primer pair was used to identify the genetic relationship among samples of this genus and to evaluate the effectiveness compared with the morphological classification method. The sequences of the two primers for detecting COI gene [20] were given as followings:

Fish F: 5′- TCAACCAACCACAAAGACATTGGCAC-3′.

Fish R: 5′ - TAGACTTCTGGGTGGCCAAAGAATCA-3′.

The sequences were used to amplify the COI gene by PCR (Polymerase Chain Reaction) method. PCR reaction was performed in a total volume of 50 μL, consisting of the components of 20 μl My Taq mix buffer 1X; 1 μl of each primer (0.25 × 10^− 6^ mol/l); 3 μl DNA corresponding to about 100 ng; and distilled water (remaining volume equivalent to 25 μl).

Polymerase chain reaction conditions were one cycle at 95 °C (2 min), 39 cycles at 94 °C (30s), 40s at the annealing temperature of 52 °C, and 1.5 min at 72 °C, and a final extension at 72 °C for 10 min. The PCR products were then sent to Macrogen Ltd. Company, the Republic of Korea, for sequencing using the method of Sanger, Nicklen and Coulson [21].

### Data analysis

The normal distribution of morphometric ratios (ED/HL, DE/HL, BD/TL, HL/TL) was tested by the Kolmogorov-Smirnov test with a sample size greater than 30 [22]. Thereafter, the Kruskal-Wallis Test was applied to analyze them if they were not normally distributed. On the contrary, the one-way ANOVA with Turkey post hoc test was used to test the spatial variation of these meristic parameters.

If morphometric ratios showed differences between species and sampling sites, the principal component analysis (PCA) was applied to determine which environmental factors and morphological characteristics (ED, DE, BD, HL, TL, W, ED/HL, DE/HL, BD/TL, HL/TL) were the main factors affecting these differences. PCA was run by PRIMER v.6 software.

Three gobioid species were identified from collected samples, comprising *G. aureus*, *G. sparsipapillus*, and *G. giuris*. The COI sequences of *G. giuris* from Australia (MW574775) and India with accession number of MK714087, MK902713, MK348190, whereas *G. aureus* from the Philippines (KJ013044), all were used as in-group controls. Two mtCOI sequences of *Butis koilomatodon* in Vietnam (OK076879) and *Periophthalmus chrysopilos* in Bangladesh (MK572461) were the out-group controls.

The obtained COI sequences with Querry ID (Table 1) were aligned in Bioedit v7.2 [23]. The genetic distances amongst three *Glossogobius* species were performed following the Kimura 2-parameters method in Mega 7.0. The genetic relationship of the twelve collected gobies specimens was identified by the “Maximum Likelihood method” with a bootstrapped value of 1000 times and performed by Mega 7.0 software [24]. “Maximum likelihood” is a the commonly used method to construct the phylogenetic tree and is used by many molecular biologists [25, 26, 27].

**Table 1:** External morphological characteristics of three species in *Glossogobius*

Species	Body color and shape	Body shape	Number of predorsal fin scales	Number of sensory papillae rows	Presence of vertical papillae rows	Presence of black spots	Presence of longitudinal black lines	Presence of vertical black bars
*Glossogobius aureus* (Akihito & Meguro, 1975)	Brownish.	Elongated.	22–27	6, on the cheek.		On the 2nd dorsal fin and the caudal peduncle.	On the side of the body but was usually blurred.	On the caudal fin.
*Glossogobius giuris* (Hamilton, 1822)	Brownish yellow.	Elongated, slender, compressed laterally.	22	10, on the cheek.	On the operculum.	Along the midline, on first dorsal-fin spine and the caudal peduncle.	On the side of body.	On the caudal fin.
*Glossogobius sparsipapillus* (Akihito & Meguro, 1976)	Brownish or yellowish.	Slender and moderate.	20–21	5–6, on the cheek.	On the operculum include the middle.	On the gill cover. On dorsal and caudal fins.		On the caudal fin.

## Results

### Species identification using morphologies

A total of 742 *G. aureus*, 1291 *G. giuris* and 764 *G. sparsipapillus* collected in the Mekong Delta were classified based on their morphological characteristics described in Table 1. *Glossogobius sparsipapillus* differed from *G. aureus* and *G. giuris* in that it had a vertical transverse of sensory papillae in the middle operculum. In the case of the distinction between *G. aureus* and *G. giuris*, the amount of predorsal scale of *G. aureus* (22–27) was greater than that of *G. giuris* (22). The values of W, TL, ED, DE, HL and BD were different from site to site (Table 2); thus, the ratios of morphometric were considered site-specific. Specially, as expressed in Table 3 (raw data can be found in supplementary material: Raw data *Glossogobius* genus), the statistical results of measurement ratios showed that *G. giuris* was different from the other two congeners. Meanwhile, *G. aureus* and *G. sparsipapillus* were statistically similar in all morphometrics traits. Namely, ED/HL and DE/HL of *G. giuris* were greater than *G. aureus* and *G. sarsipapillus*, while the opposite results were found in HL/TL and BD/TL.

**Table 2:** Variation in body measurement of three *Glossogobius* species among sampling sites

Species	Sites	n	*W* ± SE	*TL* ± SE	*ED* ± SE	*DE* ± SE	*BD* ± SE	*HL* ± SE	*ED/HL* ± SE	*DE/HL* ± SE	*HL/TL* ± SE	*BD/TL* ± SE	*BD/TL* ± SE	*HL/TL* ± SE
*Glossogobius giuris*	CRCT	310	12.63 ± 0.59	10.74 ± 0.15	0.41 ± 0.006	0.28 ± 0.008	1.37 ± 0.03	2.62 ± 0.04	0.16 ± 0.001^a^	0.11 ± 0.002^a^	0.25 ± 0.007^c^	0.13 ± 0.004^a^	0.13 ± 0.004^a^	0.25 ± 0.007^c^
	LPST	300	11.02 ± 0.64	12.57 ± 2.68	0.37 ± 0.006	0.56 ± 0.028	1.68 ± 0.04	2.07 ± 0.06	0.20 ± 0.004^b^	0.40 ± 0.026^b^	0.21 ± 0.004^a^	0.18 ± 0.005^b^	0.18 ± 0.005^b^	0.21 ± 0.004^a^
	HBBL	306	11.04 ± 0.51	10.62 ± 0.34	0.38 ± 0.005	0.32 ± 0.019	1.35 ± 0.03	2.41 ± 0.04	0.17 ± 0.003^a^	0.17 ± 0.015^a^	0.23 ± 0.002^b^	0.13 ± 0.003^a^	0.13 ± 0.003^a^	0.23 ± 0.002^b^
	DDCM	375	9.60 ± 0.32	12.65 ± 3.08	0.37 ± 0.004	0.50 ± 0.022	1.58 ± 0.03	2.12 ± 0.04	0.19 ± 0.004^b^	0.34 ± 0.021^b^	0.22 ± 0.005^ab^	0.17 ± 0.004^b^	0.17 ± 0.004^b^	0.22 ± 0.005^ab^
Kruskal-Wallis Test		χ^2^							52.35	83.15	123.99	71.93	71.93	123.99
		*p*							< 0.001	< 0.001	< 0.001	< 0.001	< 0.001	< 0.001
*Glossogobius aureus*	CRCT	166	17.45 ± 0.81	12.63 ± 0.20	0.49 ± 0.01	0.40 ± 0.009	1.57 ± 0.03	3.13 ± 0.05	0.16 ± 0.003^b^	0.13 ± 0.003^c^	0.25 ± 0.001^b^	0.12 ± 0.001^ab^	0.12 ± 0.001^ab^	0.25 ± 0.001^b^
	LPST	165	12.36 ± 0.54	11.12 ± 0.19	0.42 ± 0.008	0.40 ± 0.012	1.46 ± 0.03	2.79 ± 0.06	0.15 ± 0.002^b^	0.14 ± 0.003^d^	0.25 ± 0.002^b^	0.13 ± 0.001^b^	0.13 ± 0.001^b^	0.25 ± 0.002^b^
	HBBL	194	8.77 ± 0.38	9.87 ± 0.13	0.36 ± 0.007	0.23 ± 0.007	1.22 ± 0.02	2.34 ± 0.04	0.15 ± 0.002^b^	0.09 ± 0.002^a^	0.24 ± 0.002^a^	0.12 ± 0.002^a^	0.12 ± 0.002^a^	0.24 ± 0.002^a^
	DDCM	217	13.54 ± 0.86	11.07 ± 0.18	0.37 ± 0.007	0.28 ± 0.01	1.34 ± 0.03	2.59 ± 0.05	0.14 ± 0.002^a^	0.11 ± 0.003^b^	0.23 ± 0.002^a^	0.12 ± 0.001^a^	0.12 ± 0.001^a^	0.23 ± 0.002^a^
Kruskal-Wallis Test		χ^2^							40.04	155.16	32.39	79.92	79.92	32.39
		*p*							< 0.001	< 0.001	< 0.001	< 0.001	< 0.001	< 0.001
*Glossogobius sparsipapillus*	CRCT	159	11.53 ± 0.40	11.21 ± 0.16	0.44 ± 0.007	0.33 ± 0.011	1.40 ± 0.02	2.76 ± 0.04	0.16 ± 0.002^bc^	0.12 ± 0.003^a^	0.25 ± 0.002^bc^	0.13 ± 0.001^b^	0.13 ± 0.001^b^	0.25 ± 0.002^bc^
	LPST	196	10.21 ± 0.39	10.54 ± 0.14	0.42 ± 0.006	0.38 ± 0.008	1.28 ± 0.02	2.62 ± 0.03	0.16 ± 0.002^c^	0.14 ± 0.002^b^	0.25 ± 0.001^c^	0.12 ± 0.001^a^	0.12 ± 0.001^a^	0.25 ± 0.001^c^
	HBBL	187	9.87 ± 0.39	10.64 ± 0.16	0.39 ± 0.006	0.33 ± 0.012	1.26 ± 0.02	2.56 ± 0.04	0.15 ± 0.001^ab^	0.12 ± 0.003^a^	0.24 ± 0.001^a^	0.12 ± 0.001^a^	0.12 ± 0.001^a^	0.24 ± 0.001^a^
	DDCM	222	10.63 ± 0.39	10.55 ± 0.13	0.39 ± 0.005	0.32 ± 0.008	1.32 ± 0.02	2.56 ± 0.03	0.15 ± 0.001^a^	0.12 ± 0.002^a^	0.24 ± 0.001^ab^	0.13 ± 0.001^b^	0.13 ± 0.001^b^	0.24 ± 0.001^ab^
Kruskal-Wallis Test		χ^2^							17.92	47.02	28.56	26.28	26.28	28.56
		*p*							< 0.001	< 0.001	< 0.001	< 0.001	< 0.001	< 0.001

*W* weight (g), *TL* total length (cm), *ED* eye diameter (cm), *DE* distance between eyes (cm), *BD* body depth (cm), *HL* head length (cm), *n* number of fish use, *CRCT* Cai Rang, Can Tho, *LPST* Long Phu, Soc Trang, *HBBL* Hoa Binh, Bac Lieu, *DDCM* Dam Doi, Ca Mau

**Table 3:** The ration variation of morphometric ratios among three *Glossogobius* species

Meristic parameters	Species	Mean ± SE	Kolmogorov-Smirnov test		Kruskal-Wallis Test	
			*KS*	*P*	*χ*^*2*^	*p*
ED/HL	*Glossogobius giuris*	0.18 ± 0.002^b^	0.23	< 0.001	59.14	< 0.001
	*Glossogobius aureus*	0.15 ± 0.001^a^	0.08	< 0.001		
	*Glossogobius sparsipapillus*	0.16 ± 0.001^a^	0.03	< 0.001		
DE/HL	*Glossogobius giuris*	0.26 ± 0.010^b^	0.39	< 0.001	31.73	< 0.001
	*Glossogobius aureus*	0.12 ± 0.001^a^	0.06	< 0.001		
	*Glossogobius sparsipapillus*	0.13 ± 0.001^a^	0.07	< 0.001		
HL/TL	*Glossogobius giuris*	0.23 ± 0.002^a^	0.31	< 0.001	87.87	< 0.001
	*Glossogobius aureus*	0.24 ± 0.001^b^	0.15	< 0.001		
	*Glossogobius sparsipapillus*	0.24 ± 0.001^b^	0.16	< 0.001		
BD/TL	*Glossogobius giuris*	0.16 ± 0.002^b^	0.33	< 0.001	80.03	< 0.001
	*Glossogobius aureus*	0.12 ± 0.001^a^	0.16	< 0.001		
	*Glossogobius sparsipapillus*	0.12 ± 0.001^a^	0.14	< 0.001		

**Fig. 2:**
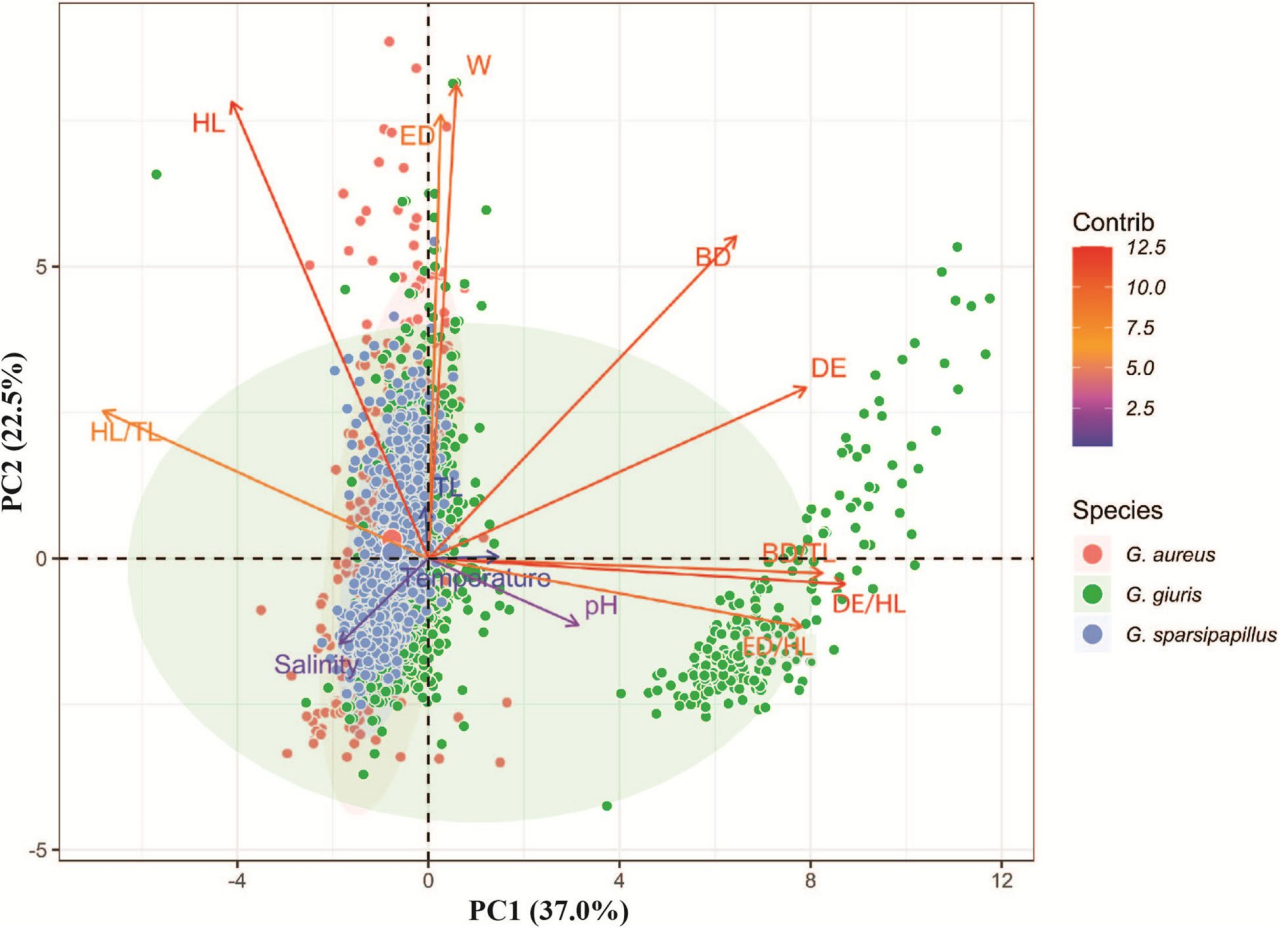
PCA plot of quantitative variables showing the correlations between the environmental factors and morphological characteristics of 3 species in *Glossogobius* genus. The orange, green and blue dots represent 742 *G. aureus*, 1291 *G. giuris* and 764 *G. sparsipapillus*, respectively

Figure 2 summarizes the results from the first exploratory multivariate analysis, PCA for 3 species in *Glossogobius* genus. In general, with 2 principal components extracted, the results explained a 59.5% of the variances (PC1: 37% and PC2: 22.5%). The principal component 1 (PC1) was the most associated with HL/TL, BD/TL, ED/HL, and DE/HL factors, whereas PC2 was the most associated with environmental factors (salinity, temperature, and pH). Figure 2 also expresses that *G. aureus* and *G. sparsipapillus* have more morphological similarities than *G. giuis* because they are superimposed in the graph while most *G. giuis* separates into two groups.

**Fig. 3:**
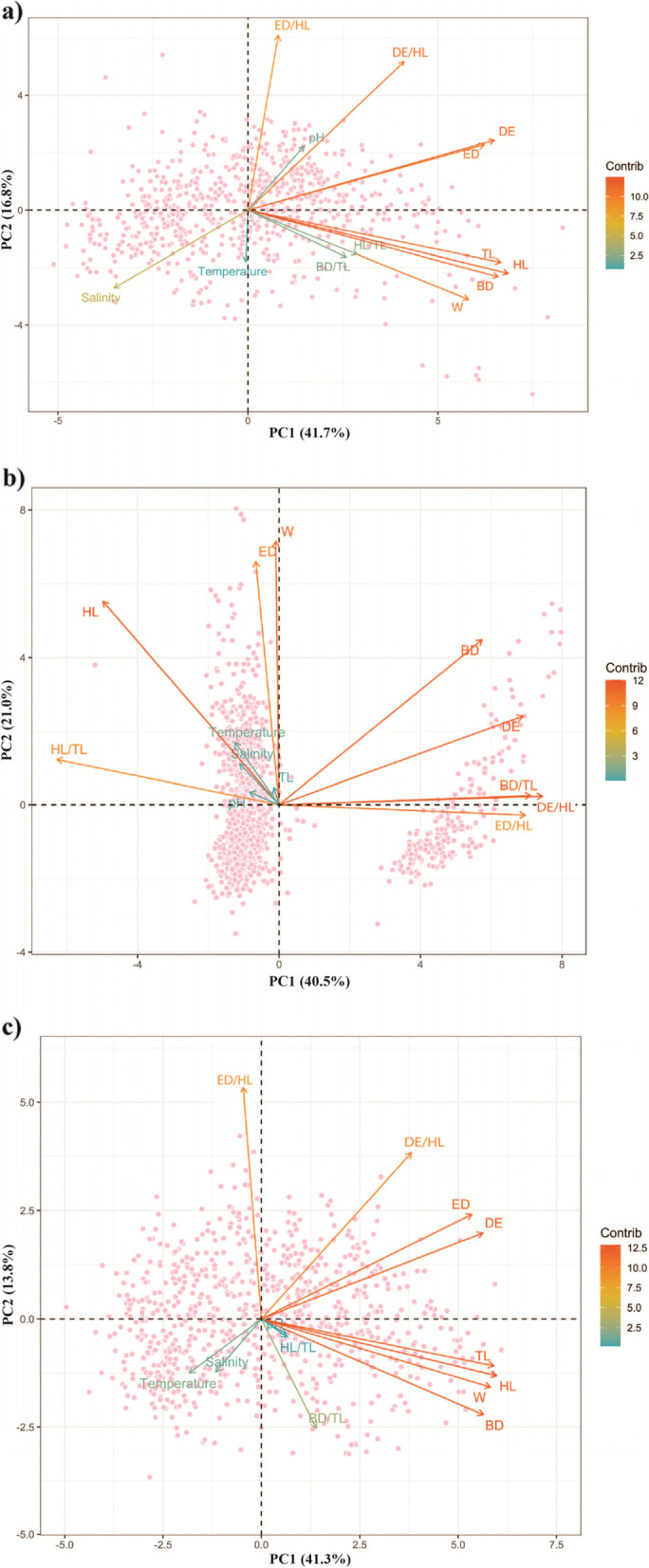
PCA plots of quantitative variables showing the correlations between environmental factors and morphological characteristics of 3 species, *G. aureus* (**a**, *n* = 742), *G. giuris* (**b**, *n* = 1291), and *G. sparsipapillus* (**c**, *n* = 764)

The principal component analysis of each species were also performed to determine the factors causing the difference between these three species. The results showed that, in all three *Glossogobius* species, the characteristic indexes of HL/TL, BD/TL, ED/HL, DE/HL and morphological measurement of HL, BD, ED, DE and W played an essential role in causing the differences amongst three species. Besides, environmental factors varied from species to species (Fig. 3). Specifically, in *G. aureus*, salinity had the most significant influence on morphological characteristics compared to temperature and pH. In the case of *G. giuris*, all three environmental factors such as salinity, temperature and pH affected the outside features, but the temperature was the strongest influencing factor, followed by pH and salinity. Whereas in the case of *G. sparsipapillus*, morphology was closely related to temperature and pH but not affected by salinity.

### Nucleotide composition

The mitochondrial cytochrome oxidase I (COI) region of all samples was successfully amplified using PCR. The sequences varied from 650 bp to 680 bp, with the composition of nucleotide presented in Table 4. There was not too much difference in the percentage of the base composition of COI sequences in *G. aureus* and *G. sparsipapillus*, namely, %T content was the highest, followed by %C, %A and the lowest was %G. A different order was observed in *G. giuris* in CRCT, LPST,HBBL and *G. sparsipapillus* in CRCT. The %C and %T were approximately the same, followed by %A and %G presented the lowest. In most cases, % AT content was always higher than %GC.

**Table 4:** Nucleotide percentage (%) of COI gene of three *Glossogobius* species

Species	Sampling sites	Accession number	%A	%C	%G	%T
*Glossogobius aureus*	Cai Rang, Can Tho	ON217530	24.50	26.67	18.60	30.23
	Long Phu, Soc Trang	OK043695	23.69	28.60	18.95	28.76
	Hoa Binh, Bac Lieu	ON217531	24.50	26.67	18.60	30.23
	Dam Doi, Ca Mau	OK043694	24.53	26.40	17.77	31.30
*Glossogobius giuris*	Cai Rang, Can Tho	OK043696	24.53	28.76	17.94	28.76
	Long Phu, Soc Trang	OK043697	24.37	28.93	17.94	28.76
	Hoa Binh, Bac Lieu	OK043698	24.37	28.93	17.94	28.76
	Dam Doi, Ca Mau	ON247043	24.37	26.57	17.60	31.47
*Glossogobius sparsipapillus*	Cai Rang, Can Tho	OK043700	24.37	28.93	17.94	28.76
	Long Phu, Soc Trang	ON217532	24.50	26.67	18.60	30.23
	Hoa Binh, Bac Lieu	ON217533	24.65	26.82	18.45	30.08
	Dam Doi, Ca Mau	OK043699	24.37	26.40	17.94	31.30

### Species identification using COI sequences

Analyzing the intraspecific alignment results of *G. aureus*, *G. giuris* and *G. sparsipapillus* by the “Align by ClustalW” method showed the variable nucleotides were 83/591; 78/591 and 79/591, respectively. In addition, the most conserved nucleotides were found in *G. giuris* (513/591), followed by *G. sparsipapillus* (512/591), and lastly *G. aureus* (508/591).

The twelve obtained COI sequences were compared to similar sequences in the Genbank by the BLAST program (Table 5) to re-identify the species. *Glossogobius giuris* - HBBL; *G.giuris* - LPST and *G. sparsipapillus* - CRCT were similar to *G. giuris* (MW574775) in Australia with 100% similarity, while 100% similarity was also seen between *G. aureus*- LPST and *G. aureus* (KJ013044) in the Philippines. There were two notable results as presented in Table 4. First, the COI gene sequence of *G. aureus* from CRCT, HBBL and DDCM showed a relative similarity with *G. giuris* (MK714087 and MK902713) from India. Second, *G. sparsipapillus* - CRCT was identical to *G. giuris* from Australia (100%), while *G. sparsipapillus* from LPST, HBBL and DDCM were only 87.25–87.48% similar to *G. giuris* prevailing from India instead of Australia.

**Table 5:** The similarity of the COI gene sequence of three *Glossogobius* species in the study with species on Gene Bank

No.	Morphology method	DNA barcoding method					
		Species	Accession number	GS (bp)	QC (%)	I (%)	Site
1	*Glossogobius aureus* - CRCT	*Glossogobius giuris*	MK714087	690	99	87.71	India
2	*Glossogobius aureus* - LPST	*Glossogobius aureus*	KJ013044	684	100	100	Philippines
3	*Glossogobius aureus* - HBBL	*Glossogobius giuris*	MK714087	690	99	87.71	India
4	*Glossogobius aureus* - DDCM	*Glossogobius giuris*	MK902713	620	100	87.48	India
5	*Glossogobius giuris* - CRCT	*Glossogobius giuris*	MW574775	598	98	99.83	Australia
6	*Glossogobius giuris* - LPST	*Glossogobius giuris*	MW574775	598	98	100	Australia
7	*Glossogobius giuris* - HBBL	*Glossogobius giuris*	MW574775	598	98	100	Australia
8	*Glossogobius giuris* - DDCM	*Glossogobius giuris*	MK902713	620	99	87.01	India
9	*Glossogobius sparsipapillus* - CRCT	*Glossogobius giuri*	MW574775	598	98	100	Australia
10	*Glossogobius sparsipapillus* - LPST	*Glossogobius giuri*	MK348190	684	99	87.42	India
11	*Glossogobius sparsipapillus* - HBBL	*Glossogobius giuri*	MK714087	690	99	87.25	India
12	*Glossogobius sparsipapillus* - DDCM	*Glossogobius giuris*	MK902713	620	100	87.48	India

*GS* gene size in bp, *QC* query cover, *I* identity, *CRCT* Cai Rang, Can Tho, *LPST* Long Phu, Soc Trang, *HBBL* Hoa Binh, Bac Lieu, *DDCM* Dam Doi, Ca Mau

### Genetic distance

The genetic distance analysis of the twelve samples of goby species was quite different. The values are presented in Table 6. Generally, “intra-species” the genetic distances of the three species in *Glossogobius* genus were similar, and ranged 0.00 to 0.12. Notably, for *G. aureus*, *G. giuris* and *G. sparsipapillus*, the “intra-species” genetic distances were similar to that observed for “inter-specific” pairwise comparisons.

**Table 6:** Genetic distances based on Kimura-2 parameters among samples of three *Glossogobius* species (Analyses were conducted using the Kimura 2-parameter model. Evolutionary analyses were conducted in MEGA7)

Samples	(1)	(2)	(3)	(4)	(5)	(6)	(7)	(8)	(9)	(10)	(11)	(12)
(1) ON217530_*G. aureus*-CRCT	_	_	_	_	_	_	_	_	_	_	_	_
(2) OK043695_*G. aureus*-LPST	0.12	_	_	_	_	_	_	_	_	_	_	_
(3) ON217531_*G. aureus*-HBBL	0.00	0.12	_	_	_	_	_	_	_	_	_	_
(4) OK043694_*G. aureus*-DDCM	0.00	0.12	0.00	_	_	_	_	_	_	_	_	_
(5) OK043696_*G. giuris*-CRCT	0.11	0.12	0.11	0.11	_	_	_	_	_	_	_	_
(6) OK043697_*G. giuris*-LPST	0.11	0.11	0.11	0.11	0.00	_	_	_	_	_	_	_
(7) OK043698_*G. giuris*-HBBL	0.11	0.11	0.11	0.11	0.00	0.00	_	_	_	_	_	_
(8) ON247043_*G. giuris*-DDCM	0.00	0.12	0.00	0.00	0.12	0.11	0.11	_	_	_	_	_
(9) OK043700_*G. sparsipapillus*-CRCT	0.11	0.11	0.11	0.11	0.00	0.00	0.00	0.11	_	_	_	_
(10) ON217532_*G. sparsipapillus*-LPST	0.00	0.12	0.00	0.00	0.11	0.11	0.11	0.00	0.11	_	_	_
(11) ON217533_*G. sparsipapillus*-HBBL	0.00	0.12	0.00	0.00	0.11	0.11	0.11	0.01	0.11	0.00	_	_
(12) OK043699_*G. sparsipapillus*-DDCM	0.00	0.12	0.00	0.00	0.11	0.11	0.11	0.00	0.11	0.00	0.00	_

*CRCT* Cai Rang, Can Tho, *LPST* Long Phu, Soc Trang, *HBBL* Hoa Binh, Bac Lieu, *DDCM* Dam Doi, Ca Mau

### Genetic relationship analysis

**Fig. 4:**
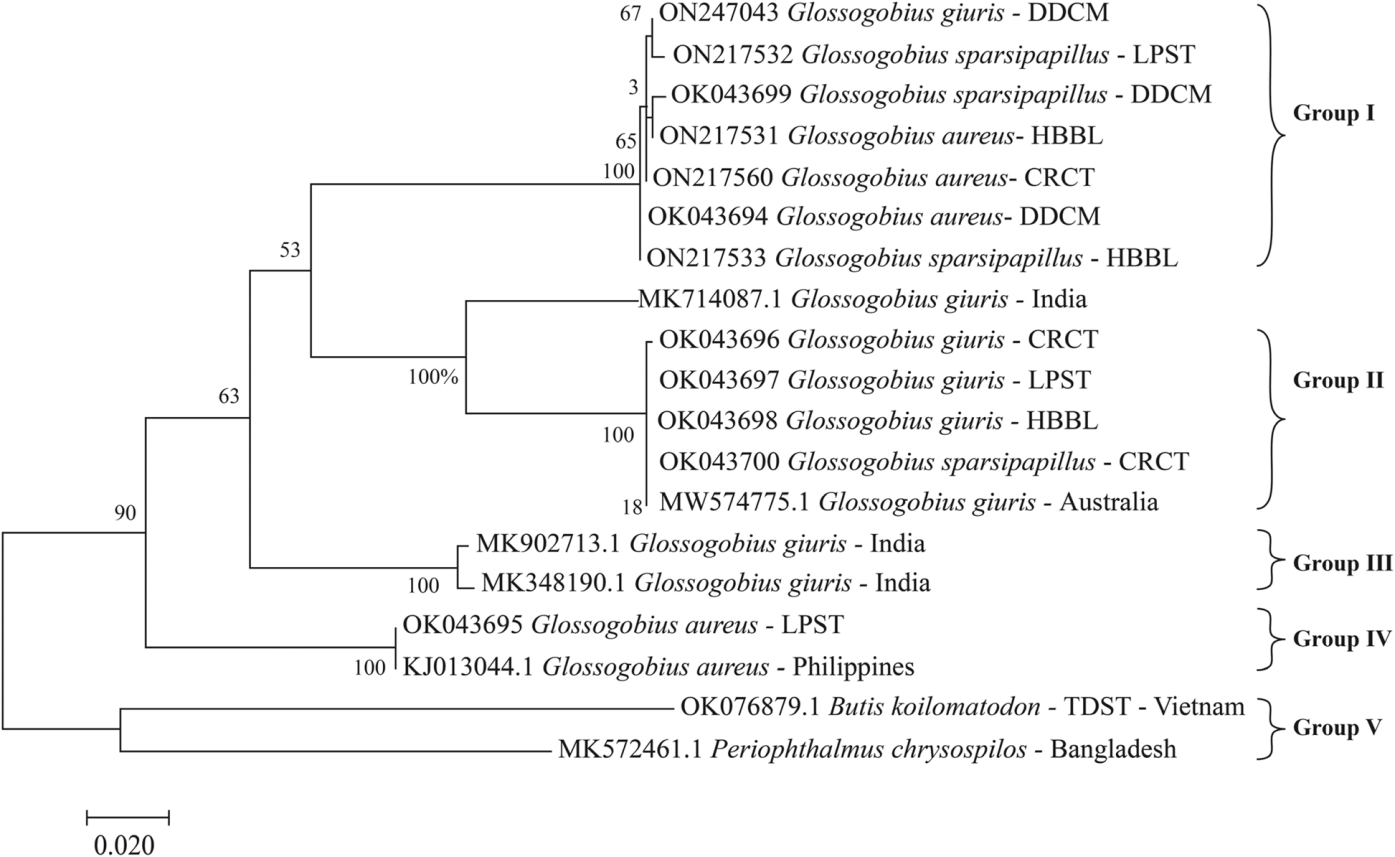
The phylogenetic tree based on the COI gene was inferred by using the Maximum Likelihood method based on the Tamura-Nei model, with a bootstrap value of 1000 times on the nodes. Branch lengths correspond to the mean number of nucleotide substitutions per site. Scale bar indicates substitutions per site

The phylogenetic tree of *G. aureus*, *G. giuris* and *G. sparsipapillus* in Fig. 4 showed that species samples had a very distinct division with a bootstrap index in many nodes as high as 100% for the effectiveness and accuracy of the species identification by the COI gene. The phylogenetic tree of the studied fish in Fig. 4 was divided into five main groups. In Group I, two *G. aureus* individuals in CRCT and HBBL were identical to *G. sparsipapillus* individuals in LPST, because the number of substitutions per site was 0.00. While *G. giuris*-DDCM and *G. aureus*-DDCM exhibited similarities to *G. sparsipapillus*-LPST and *G. sparsipapillus*-HBBL, respectively.

*Glossogobius giuris* from CRCT, LPST, and HBBL together with *G. sparsipapillus* were in group II. Compared with the in-group control sequence of *G. giuris* from Australia, they were also the same in heredity, however they expressed difference from *G. giuris* in India, due to the identical percentage of 87.01%. This showed that the *G. giuris* specimens were correctly identified but needed to be reviewed for *G. sparsipapillus*. Group III consisted two in-group controls of *G. giuris* from India, while group V was two out-group controls of *B. koilomatodon*-Vietnam and *P. chrysopilos*-Bamgladesh. Group IV included *G. aureus* - LPST and *G. aureus* from the Philippines (in-group control) with 100% similarity. Overall, the twelve selected sequences did not cluster according to the morphological attribution, being interspersed in the phylogenetic tree, which however, identified three main groups (excluding two control groups III and V), suggesting the existence of three distinct lineages.

## Discussion

This study involved species identification based on the morphology and COI sequences as DNA barcoding. The morphological descriptions of *Glossogobius* species were similar to the studies on the morphology of *G. giuris* of Herre [28] and Tran, Shibukawa, Nguyen, Ha, Tran, Mai and Utsugi [17]; the study on *G. aureus* of Phuong and Binh [29]; and the study on *G. sparsipapillus* of Tran, Shibukawa, Nguyen, Ha, Tran, Mai and Utsugi [17]. Tran, Shibukawa, Nguyen, Ha, Tran, Mai and Utsugi [17] reported that the standard length of *G. giuris*, *G. aureus* and *G. sparsipapillus* in the Mekong Delta region could reach different sizes. However, very few morphological characters alone are sufficient to identify *G. sparsipapillus*, *G. aureus* and *G. giuris*, namely the vertical transverse of sensory papillae in the middle operculum and the number of predorsal scales.

Kamboj and Kamboj [30] and Ujjania, Kumar, Langar and Krishna [31] noted that the morphometric parameters increased proportionally to the length of the fish. Meanwhile, meristic counts and meristic variables were independent of fish size, but affected by the phylogenetic origin and gender [32, 33]. In the present study, the measurement ratios of *G. giuris* were different from those of the two other species. However, whether these morphological differences were due to genetics or adaptation of the fish to the environment needed to be determined based on the study of the mtCOI gene.

There were discrepancies in the BLAST results of *Glossogobius* specimens. For example, *G. aureus* - DDCM was relatively similar to *G. giuris* (MK902713) from India, rather than *G. aureus*, which may be due to an error in the sequence. Furthermore, all *G. sparsipapillus* specimens were low homologous to *G. giuris* (MK902713) from India because the genetic data of *G. sparsipapillus* was unavailable in the Genbank. In addition, *G. sparsipapillus* - CRCT was identical to *G. giuris* from Australia (100%), while *G. sparsipapillus* - DDCM was only 87.48% similar to *G. giuris* from India instead of Australia. The inconsistency in these results may be due to the interfered nucleotides (errors in sequencing), resulting in a different intra-species genetic distance (0.11) of *G. sparsipapillus*. Therefore, it was necessary to reclassify this species by both morphology and DNA barcoding methods.

The COI sequence was reported to be informative in analyzing genetic diversity in fish, including Australian fish species [20], medicinal fish of Culter (Pisces: Cyprinidae) [34], pufferfish species [35], transparent gobies [36], and Sillaginidae fishes (Perciformes) [37]. Within the scope of this study, three *Glossogobius* species exhibited a lot of similar outside traits as mentioned by Hoese and Allen [38], *G. giuris*, *G. aureus* and *G. sparsipapillus* had a cylindrical body, with two distinct dorsal fins and fused pelvic fins. Some other features were notable, such as largemouth (10–15% SL), depressed head, long and pointed snout l, projecting lower jaw, at least six lines of longitudinal papilla running longitudinally on the cheek, 27–30 vertebrae, a bilobed tongue, gill opening reaching below a point just before to just behind posterior preopercular margin. However, *G. giuris* had 22 predorsal fin rays, a unique criterion distinguishing tank goby from two other congeners [39], and *G. sparsipapillus* had a vertical transverse of sensory papillae in the middle operculum. The fact that *G. sparsipapillus* was identical to *G. giuris* or *G. aureus* could be due to two main reasons; one was that the COI gene sequence of *G. sparsipapillus* was not available in the gene bank; and the second was that COI gene sequence of *G. sparsipapillus* still had many overlapping nucleotides at different peaks. *Glossogobius aureus* had the same characteristic documented by Hoese and Allen [38] such as the blackish spots on the 2nd dorsal fin and in the caudal peduncle, as well as the longitudinal black lines on the side of the body which are usually blurred as reported by Phuong and Binh [29]. The phylogram showed that the three species of the *Glossogobius* genus were interspersed in small clades. Moreover, the genetic distance between the three *Glossogobius* species (≤ 0.159 or 15.9%) was smaller than the average difference between species of the same genus in the suborder Butidae (22.2%) [40], but higher than between fish species in Australia (9.93%) [20]. The result showed that the difference in COI sequence of species in the *Glossogobius* genus was relatively low. As such it is recommended that it is with morphological methods or the other mitochondrial DNA barcodes such as cytochrome b, 12S or 16S rRNA to classify the species more accurately.

Previously, morphological characters were mainly used to identify fish species and phylogenetic relationships to understand their speciation and evolution [41, 42]. On the contrary, gobiid species were hard to differentiate species because of their similarity in external morphology [4, 43]. Therefore, the reconstructed phylogenetic trees based on morphology were limited, and at times controversial due to the complex evolutionary changes in either morphological or physiological characters [44, 45]. Based on the development of molecular biology techniques, this situation has changed, especially with the application of mtDNA’s genetic analysis to resolve controversial taxonomic problems [46–49]. This technique is a helpful tool for the determination of molecular markers that can facilitate the discrimination of morphologically similar species. Many previous researchers studied the gobiid fishes and reported that they are monophyletic [42, 44, 45, 50]. In the present research, the COI part of the mitochondrial DNA was sequenced to identify gobies from twelve samples collected from different provinces in the Vietnamese Mekong Delta. Nevertheless, the COI sequence of gobies displayed a similarity to available sequences of the gene bank. As taxonomic ambiguities, successful molecular identification was helpful. The findings showed that the COI gene enabled accurate fish species identification where adequate sequence data exists.

## Conclusion

There was an incongruence between morphological and molecular species attribution between morphological and molecular species attribution of three species: *G. aureus*, *G. giuris* and *G. sparsipapillus* collected in brackish and freshwater in the Mekong Delta based on the COI gene sequences. Morphological characteristics and fish body size of *G. aureus* and *G. sparsipapillus* had many similarities in the present study such as ED/HL, DE/HL, HL/TL and BD/TL, while *G. giuris* showed more differences. Their COI sequences were similar up to100% to species in the *Glossogobius* genus on NCBI. Despite the differences in their morphometric characteristics, *G. aureus*, *G. giuris* and *G. sparsipapillus* were nearly genetically identical up 99–100%. Thus, further research was needed to reclassify *Glossogobius* species in VDM to contribute to developing a conservation strategy for these economically valued species.

## Supplementary Information

**Additional file 1.**

**Additional file 2.**

**Additional file 3.**

**Additional file 4.**

## Data Availability

All data generated or analyzed during this study are included in this published article [and its supplementary information file, namely Raw data_*Glossogobius* genus]. The sequences of the COI gene were submitted to NCBI and got the ID: OK043694-OK043700 (please find the GenBank_OK043694-OK043700 file submitted to the Journal system].

## Abbreviations

*COI*: *Cytochrome c oxidase* subunit I
VMD: The Mekong Delta, Vietnam

## References

[1] Le T, Nguyen MT, Nguyen VP, Nguyen DC, Pham XH, Nguyen TS, Hoang VC, Hoang PL, Le H, Dao NC (2006). Provinces and City in the Mekong Delta. Geography of Provinces and Cities in Vietnam. Edited by Le T, vol. VI.

[2] Diep AT, Dinh QM, Tran DD (2014). Species composition of gobiidae distributed in the coastal areas, Soc Trang Province. VNU J Sci.

[3] Tran DD, Nguyen VT, To HTM, Nguyen TT, Dinh QM (2020). Species composition and biodiversity index of gobiid assemblage in estuarine areas of the Mekong Delta, Vietnam. EJABF.

[4] Dinh QM, Tran DD, Vo TT, Nguyen MT, Phan NY (2018). Study on species composition and some biodiversity indices of gobies distributing in the muddy flat along the coastline in the Mekong Delta.

[5] Dinh QM (2008). Data of survey on the species composition of fishes in Hau Basin at an Phu district, an Giang province. Can Tho Univ J Sci.

[6] Dinh QM, Pham TT, Nguyen KTL (2009). Preliminary data on the species composition fish in Co Chien and Ham Luong river basins at Mo Cay District, Ben Tre Province. Proceedings of the 3rd National Scientific Conference on Ecology and Biological Resources.

[7] Nguyen THD, Nguyen HTT, Tran TC, Nguyen YTN, Dinh QM (2020). Morphometric and meristic variations of Glossogobius sparsipapillus along the coastline in the Mekong Delta, Vietnam. Int J Zool Anim Biol.

[8] Phan GH, Dinh QM, Truong NT, Nguyen THD (2021). Variation in morphometric characteristics of Glossogobius aureus distributed from Can Tho to Ca Mau. Vietnam Agric Sci J.

[9] Nguyen THD, Dinh QM (2021). Morphometric and meristic variations in Glossogobius giuris distributed in different locations in the Mekong Delta. TNU J Sci Technol.

[10] Dinh QM, Nguyen THD, Nguyen TTK (2021). Allometry variation in morphometrics of Glossogobius sparsipapillus caught along Hau river, from Can Tho to Soc Trang provinces. TNU J Sci Technol.

[11] Dinh QM (2011). The species composition and distributive characteristics of Perciformes in Hau river basin in Can Tho city, Vietnam. J Sci Hanoi Univ Educ.

[12] Tran DD, Cao HV, Dinh QM, Tran LX (2020). An assessment of fisheries resources in the coastal water of the Mekong Delta, Vietnam. AACL Bioflux.

[13] Tran DD, Le BP, Dinh QM, Duong NV, Nguyen TT (2021). Fish species composition variability in cu Lao dung, Soc Trang, Vietnam. AACL Bioflux.

[14] Dinh QM, Nguyen YTN, Dang TH, Tran NS, Lam TTH, Mai HTH, Hoang NT (2019). Fish species in rice field canals in inside and outside dikes in tri ton, Cho Moi, Chau Phu, an Giang province. Dong Thap Univ Sci.

[15] Dinh QM (2018). Aspects of reproductive biology of the red goby Trypauchen vagina (Gobiidae) from the Mekong Delta. J Appl Ichthyol.

[16] Tran DD, Nguyen TV, To TMH, Nguyen TT, Dinh MQ (2020). Species composition and biodiversity index of gobiid assemblage in estuarine areas of the Mekong Delta, Vietnam. EJABF.

[17] Tran DD, Shibukawa K, Nguyen TP, Ha PH, Tran XL, Mai VH, Utsugi K (2013). Fishes of Mekong Delta, Vietnam.

[18] Rogers SO, Bendich AJ (1989). Extraction of DNA from plant tissues. Plant molecular biology manual.

[19] Nguyen TP, Duong YT (2015). Comparing morphological characteristics and DNA barcoding of two goby species Butis butis and Butis humeralis. Can Tho Univ J Sci.

[20] Ward RD, Zemlak TS, Innes BH, Last PR, Hebert PD (2005). DNA barcoding Australia's fish species. PhilosTransact R Soc B.

[21] Sanger F, Nicklen S, Coulson AR (1977). DNA sequencing with chain-terminating inhibitors. Proc Natl Acad Sci.

[22] Drezner Z, Turel O, Zerom D (2010). A modified Kolmogorov–Smirnov test for normality. Commun Stat Simul Comput.

[23] Hall T. BioEdit: a user-friendly biological sequence alignment editor and analysis program for windows 95/98/NT. Nucleic Acids Symp Ser. 1999;41(41):95–8.

[24] Kumar S, Stecher G, Tamura K (2016). MEGA7: molecular evolutionary genetics analysis version 7.0 for bigger datasets. Mol Biol Evol.

[25] Agorreta A, Rueber L (2012). A standardized reanalysis of molecular phylogenetic hypotheses of Gobioidei. Syst Biodivers.

[26] Agorreta A, San Mauro D, Schliewen U, Van Tassell JL, Kovačić M, Zardoya R, Rüber L (2013). Molecular phylogenetics of Gobioidei and phylogenetic placement of European gobies. Mol Phylogenet Evol.

[27] Çiftci Y, Eroğlu O, Firidin Ş (2013). Mitochondrial cytochrome b sequence variation in three Sturgeon species (A. stellatus Pallas, 1771, A. gueldenstaedtii Brandt, 1833, H. huso Linnaeus, 1758) from the Black Sea coasts of Turkey. Turkish J Fish Aquat Sci.

[28] Herre AW (1927). Gobies of the Philippines and the China Sea. Monogr Bur Sci Manila.

[29] Phuong TTL, Binh DT (2015). Goby species diversity in Vietnam based on morphological and genetic characteristics. J Fish Sci Technol.

[30] Kamboj N, Kamboj V (2019). Morphometric and meristic study of four freshwater fish species of river ganga. Indian J Anim Sci.

[31] Ujjania N, Kumar G, Langar R, Krishna G (2012). Biometric studies of mahseer (Tor tor Ham. 1822) from Bari talab (Udaipur), India. Int Res J Biol Sci.

[32] Talwar PK, Jhingran AG (1991). Inland fishes of India and adjacent countries, vol. 2.

[33] Gonzalez-Martinez A, Lopez M, Molero HM, Rodriguez J, González M, Barba C, García A (2020). Morphometric and meristic characterization of native Chame fish (Dormitator latifrons) in Ecuador using multivariate analysis. Animals.

[34] Xie J-Y, Li J-D, Huang Y-S (2013). DNA barcoding application of mitochondrial COI gene sequence in medicinal fish of Culter (Pisces: Cyprinidae). Zhongguo Zhong Yao Za Zhi.

[35] Turan C, Gürlek M, Ergüden D, Uyan A, Karan S, Doğdu SA (2017). Assessing DNA barcodes for identification of pufferfish species (Tetraodontidae) in Turkish marine waters. Nat Eng Sci.

[36] Roesma DI, Tjong DH, Aidil DR (2020). Phylogenetic analysis of transparent gobies in three Sumatran lakes, inferred from mitochondrial cytochrome oxidase I (COI) gene. Biodivers J Biol Divers.

[37] Cheng J, Xiao J, Song N, Saha S, Qin J, Nomura H, Panhwar SK, Farooq N, Shao K, Gao T (2021). Molecular phylogeny reveals cryptic diversity and swim bladder evolution of Sillaginidae fishes (Perciformes) across the indo-West Pacific Ocean. Divers Distrib.

[38] Hoese DF, Allen GR (2009). Description of three new species of Glossogobius from Australia and New Guinea. Zootaxa.

[39] Akihito P, Meguro K (1975). Description of a new gobiid fish, Glossogobius aureus, with notes on related species of the genus. Japan J Ichthyol.

[40] Viswambharan D, Pavan-Kumar A, Singh DP, Jaiswar A, Chakraborty S, Nair JR, Lakra W (2015). DNA barcoding of gobiid fishes (Perciformes, Gobioidei). Mitochondrial DNA.

[41] Nelson J, Grande T, Wilson M (2016). Fishes of the world.

[42] Thacker CE (2003). Molecular phylogeny of the gobioid fishes (Teleostei: Perciformes: Gobioidei). Mol Phylogenet Evol.

[43] Thacker C, Patzner RA, Tassell JLV, Kovacic M, Kapoor BG (2011). Systematics of gobiies. The biology of gobies.

[44] Thacker CE (2013). Phylogenetic placement of the European sand gobies in Gobionellidae and characterization of gobionellid lineages (Gobiiformes: Gobioidei). Zootaxa.

[45] Thacker C (2009). Phylogeny of Gobioidei and placement within acanthomorpha, with a new classification and investigation of diversification and character evolution. Copeia.

[46] Rüber L, Britz R, Zardoya R (2006). Molecular phylogenetics and evolutionary diversification of labyrinth fishes (Perciformes: Anabantoidei). Syst Biol.

[47] Erguden D, Gurlek M, Yaglioglu D, Turan C (2010). Genetic identification and taxonomic relationship of Mediterranean mugilid species based on mitochondrial 16S rDNA sequence data. J Anim Vet Adv.

[48] Yang L, Tan Z, Wang D, Xue L (2014). Guan M-x, Huang T, Li R: species identification through mitochondrial rRNA genetic analysis. Sci Rep.

[49] Parmaksiz A (2021). Determination of genetic variations by using mitochondrial DNA cyt b sequences in populations of Carasobarbus luteus (Cyprinidae). Aquatic Research.

[50] Kartavtsev YP, Sharina SN, Goto T, Rutenko OA, Zemnukhov VV, Semenchenko AA, Pitruk DL, Hanzawa N (2009). Molecular phylogenetics of pricklebacks and other percoid fishes from the sea of Japan. Aquat Biol.

