# Correction: The mismatch between morphological and molecular attribution of three *Glossogobius* species in the Mekong Delta

**DOI:** 10.1186/s40850-022-00140-x

**Published:** 2022-07-22

**Authors:** Ngon T. Truong, Gieo H. Phan, Tran T. H. Lam, Ton H. D. Nguyen, Do T. Khang, Men T. Tran, Nam S. Tran, Quang M. Dinh

**Affiliations:** 1 Department of Molecular Biotechnology, Biotechnology Research and Development Institute, Can Tho University, Xuan Khanh Ward, Ninh Kieu District, Can Tho, 900000 Vietnam; 2 Faculty of Agriculture and Rural Development, Kien Giang University, Minh Luong Town, Chau Thanh District, Kien Giang 920000 Vietnam; 3 Institute of High Quality Biotechnology - Food Technology, Cuu Long University, National Road 1A, Phu Quoi Ward, Long Ho District, Vinh Long, 850000 Vietnam; 4 Department of Biology, School of Education, Can Tho University, Xuan Khanh Ward, Ninh Kieu District, Can Tho, 900000 Vietnam; 5 Department of Biology, College of Natural Science, Can Tho University, Xuan Khanh Ward, Ninh Kieu District, Can Tho, 900000 Vietnam; 6 Department of Environmental Sciences, College of Environment and Natural Resources, Can Tho University, Can Tho, 900000 Vietnam

**Correction: BMC Zool 7, 34 (2022)**

**https://doi.org/10.1186/s40850-022-00137-6**

Following publication of the original article [1], the authors reported that Table 1 looks fine on the website, but the text in columns 1-3 are covered by fish photos on the PDF version.

**Table 1:**
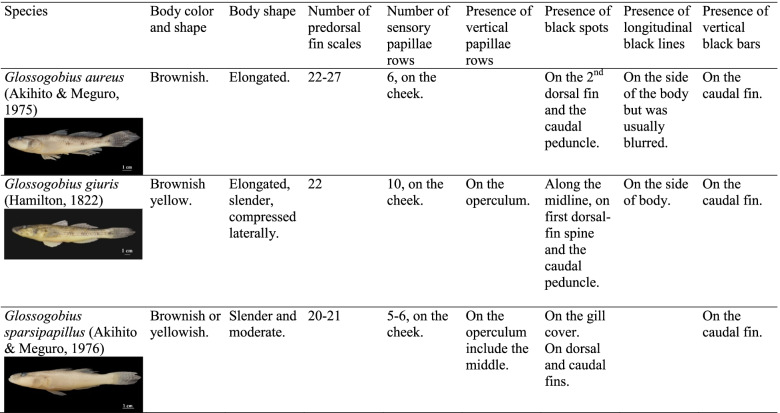
External morphological characteristics of three species in *Glossogobius*

The correct Table 1 has been provided in this Correction.

The original article [1] has been corrected.
