# Supplementary material for: The mismatch between morphological and molecular attribution of three Glossogobius species in the Mekong Delta
Source: BMC Zool. 2022 Jun 23;7:34. doi: 10.1186/s40850-022-00137-6 (PMC10126994; doi:10.1186/s40850-022-00137-6)
Supplement: Supplementary file 2 — Additional file 2. [file 40850_2022_137_MOESM2_ESM.pdf]

**Lâm Thị Huyền Trân <lamthihuyentran@mku.edu.vn>**

# GenBank ON247043

1 thư

**gb-admin@ncbi.nlm.nih.gov** <gb-admin@ncbi.nlm.nih.gov> 11:48 16 tháng 4, 2022
Tới: congthanhvl@gmail.com, lamthihuyentran@mku.edu.vn

Dear GenBank Submitter:

We have provided GenBank accession number(s) for your nucleotide sequence(s):

SUB11340643 Seq1 ON247043

A copy of your revised files can be viewed at

https://submit.ncbi.nlm.nih.gov/subs/?search=SUB11340643

The accession number link in the Submission Portal will not be active until a few days after the public release of the sequences.

Changes may have been made to your original submission in order to conform to database annotation conventions. See the following for a list of possible modifications:
https://ncbi.nlm.nih.gov/genbank/flatfile_changes/

If you need to revise your record(s), follow these directions to format your update request: https://www.ncbi.nlm.nih.gov/Genbank/update.html
Send properly formatted updates to: gb-admin@ncbi.nlm.nih.gov
Do not make a new submission for an update request.

Based on the data submitted to us, the scheduled release date for your submission is:

Apr 21, 2022

The entire sequence will be released when the article citing this accession number(s) is published or on the above release date, whichever comes first. If this date is not correct, please let us know as soon as possible, otherwise this submission will be released on the date indicated above. The data will become available from our different servers within a few days of release and are simultaneously made available to other INSDC databases, the European Nucleotide Archive (ENA) and the DNA Data Bank of Japan (DDBJ).

Thank you for your submission of sequence data to GenBank, a contribution which will benefit the scientific community.

Sincerely,

GenBank Direct Submission Staff
gb-admin@ncbi.nlm.nih.gov
