# Supplementary material for: The mismatch between morphological and molecular attribution of three Glossogobius species in the Mekong Delta
Source: BMC Zool. 2022 Jun 23;7:34. doi: 10.1186/s40850-022-00137-6 (PMC10126994; doi:10.1186/s40850-022-00137-6)
Supplement: Supplementary file 3 — Additional file 3. [file 40850_2022_137_MOESM3_ESM.pdf]

TRƯỜNG ĐẠI HỌC CỬU LONG
MEKONG UNIVERSITY

Lâm Thị Huyền Trân <lamthihuyentran@mku.edu.vn>

GenBank OK043694-OK043700

3 thư

gb-admin@ncbi.nlm.nih.gov <gb-admin@ncbi.nlm.nih.gov> 01:00 8 tháng 9, 2021
Tới: congthanhvl@gmail.com, lamthihuyentran@mku.edu.vn

Dear GenBank Submitter:

Thank you for your direct submission of sequence data to GenBank. We have provided GenBank accession number(s) for your nucleotide sequence(s):

SUB10298572 Seq1	OK043694
SUB10298572 Seq2	OK043695
SUB10298572 Seq3	OK043696
SUB10298572 Seq4	OK043697
SUB10298572 Seq5	OK043698
SUB10298572 Seq6	OK043699
SUB10298572 Seq7	OK043700

GenBank accession numbers should appear in any publication that reports or discusses the data, as it gives the community a unique label with which they may retrieve your data from our online servers.

Based on the data submitted to us, the scheduled release date for your submission is:

Jan 1, 2022

The entire sequence will be released when the article citing this accession number(s) is published or on the above release date, whichever comes first. If this date is not correct, please let us know as soon as possible, otherwise this submission will be released on the date indicated above. The data will become available from our different servers within a few days of release and are simultaneously made available to ENA in Europe and the DNA Data Bank of Japan.

A copy of your revised files can be viewed at

https://submit.ncbi.nlm.nih.gov/subs/?search=SUB10298572

The accession number link in the Submission Portal will not be active until a few days after the public release of the sequences.

Changes may have been made to your original submission in order to conform to database annotation conventions including:

- Strings of N's, low quality sequence, vector or linker trimmed from ends
- Feature spans corrected and descriptions modified for all feature types including coding regions
- Nomenclature edited to 'official' gene names, product labels, etc.
- Exon spans adjusted to conform to the splice donor/acceptor consensus sequences, GT and AG, respectively
- Any mRNA or ribosomal RNA sequences submitted on the minus-strand have been reverse-complemented
- Taxonomic and source data edited, including unpublished organism names changed to temporary names. Please notify us when the organism names

are published and we will update them accordingly.

If you have included voucher, culture collection or biomaterial information within your submission, the controlled list of institution codes can be found at: ftp://ftp.ncbi.nlm.nih.gov/pub/taxonomy/coll_dump.txt If the codes in your submission have been interpreted incorrectly, please notify us.

If your submission needs revision, do not submit a new sequence. Instead, please follow the directions to update a sequence record at: https://www.ncbi.nlm.nih.gov/Genbank/update.html Since the flatfile record is a display format only and is not an editable format of the data, do not make changes directly to a flatfile. Send properly formatted updates to: gb-admin@ncbi.nlm.nih.gov

Sincerely,

GenBank Direct Submission Staff
gb-admin@ncbi.nlm.nih.gov

Lâm Thị Huyền Trân <lamthihuyentran@mku.edu.vn> 07:22 8 tháng 9, 2021
Tới: dmquang@ctu.edu.vn

Được gửi từ iPhone của tôi

Bắt đầu thư được chuyển tiếp:

Từ: gb-admin@ncbi.nlm.nih.gov
Ngày: 01:01:04 GMT+7 ngày 8 tháng 9, 2021
Đến: congthanhvl@gmail.com, congthanhvl@gmail.com, lamthihuyentran@mku.edu.vn
Chủ đề: GenBank OK043694-OK043700

[Ẩn văn bản trích dẫn]

Lâm Thị Huyền Trân <lamthihuyentran@mku.edu.vn> 12:21 8 tháng 9, 2021
Tới: gb-admin@ncbi.nlm.nih.gov

Dear GenBank Direct Submission Staff,
On behalf of the research team, I would like to express my sincere gratitude to GenBank Direct Submission Staff for your warnings and dedicated instructions to help me alter errors in the sequences I uploaded. We have submitted the article related to these sequences to Egyptian Journal of Aquatic Research, and are editing it as requested by the editors and reviewers. We will notify you as soon as the article will be published.
Best regards.
Tran Lam.

Vào Th 4, 8 thg 9, 2021 vào lúc 01:01 <gb-admin@ncbi.nlm.nih.gov> đã viết:
[Ẩn văn bản trích dẫn]
